# Gastrin-induced miR-222 promotes gastric tumor development by suppressing p27^kip1^

**DOI:** 10.18632/oncotarget.9990

**Published:** 2016-06-14

**Authors:** Katie A. Lloyd, Andrew R. Moore, Bryony N. Parsons, Adrian O'Hara, Malcolm Boyce, Graham J. Dockray, Andrea Varro, D. Mark Pritchard

**Affiliations:** ^1^ Department of Cellular and Molecular Physiology, Institute of Translational Medicine, University of Liverpool, Liverpool, United Kingdom; ^2^ Gastroenterology Directorate, Royal Liverpool and Broadgreen University Hospitals NHS Trust, Liverpool, United Kingdom; ^3^ Trio Medicines Ltd, London, United Kingdom

**Keywords:** neuroendocrine, carcinoid, microRNA, stomach, CCK2 receptor

## Abstract

**Background and Aims:**

Elevated circulating concentrations of the hormone gastrin contribute to the development of gastric adenocarcinoma and types-1 and 2 gastric neuroendocrine tumors (NETs). MicroRNAs (miRNAs) are small non-coding RNAs that post-transcriptionally regulate proteins which in turn influence various biological processes. We hypothesised that gastrin induces the expression of specific gastric miRNAs within CCK2 receptor (CCK2R) expressing cells and that these mediate functionally important actions of gastrin.

**Results:**

Gastrin increased miR-222 expression in AGS_GR_ cells, with maximum changes observed at 10 nM G17 for 24 h. Signalling occurred via CCK2R and the PKC and PI3K pathways. miR-222 expression was increased in the serum and gastric corpus mucosa of hypergastrinemic INS-GAS mice and hypergastrinemic patients with autoimmune atrophic gastritis and type 1 gastric NETs; it decreased in patients following treatment with the CCK2R antagonist netazepide (YF476). Gastrin-induced miR-222 overexpression resulted in reduced expression and cytoplasmic mislocalisation of p27^kip1^, which in turn caused actin remodelling and increased migration in AGS_GR_ cells.

**Materials and Methods:**

miRNA PCR arrays were used to identify changes in miRNA expression following G17 treatment of human gastric adenocarcinoma cells stably transfected with CCK2R (AGS_GR_). miR-222 was further investigated using primer assays and samples from hypergastrinemic mice and humans. Chemically synthesised mimics and inhibitors were used to assess cellular phenotypical changes associated with miR-222 dysregulation.

**Conclusions:**

These data indicate a novel mechanism contributing to gastrin-associated gastric tumor development. miR-222 may also be a promising biomarker for monitoring gastrin induced premalignant changes in the stomach.

## INTRODUCTION

Elevated circulating concentrations of the gastric antral hormone gastrin are found in patients who are hypochlorhydric as a result of atrophic gastritis. Autoimmune atrophic gastritis predisposes to the development of type 1 gastric neuroendocrine (carcinoid) tumors (NETs) [1, 2]. Hypergastrinemia is crucial for the development of these tumors, as surgical antrectomy to remove the source of hypergastrinemia [3] or treatment with gastrin/CCK2 receptor antagonist drugs can reverse tumor development [4, 5]. *Helicobacter pylori* induced atrophic gastritis also results in hypergastrinemia and this is thought to act as a co-factor during gastric adenocarcinoma development. This is supported by animal studies which have demonstrated accelerated *H. pylori* induced gastric carcinogenesis in transgenic hypergastrinemic INS-GAS mice [6, 7].

Gastrin contributes to gastric tumor development via several cellular mechanisms. These are in addition to its well established role in regulating gastric acid secretion and include alterations in cell proliferation, apoptosis, migration, differentiation and angiogenesis (reviewed in [8–10]). Moreover several proteins including Reg [11], MMP-7 [12], MMP-1 [13] and members of the urokinase plasminogen activator family of proteins [14] show increased expression in the stomach or serum of patients with hypergastrinemia. Many of these proteins are thought to contribute to gastric tumorigenesis by altering key functions including cell migration and differentiation. Some may also have utility as biomarkers of tumor development. Gastrin exerts its effects in the stomach predominantly as a result of binding to the CCK2 receptor (CCK2R) on enterochromaffin-like (ECL) cells. Downstream signalling occurs via a number of pathways, including protein kinase C (PKC), MAP kinase (MAPK), and phosphatidylinositol (PI) 3-kinase (PI3K) [8, 9].

MicroRNAs (miRNAs) are a class of endogenous non-protein coding short RNAs that post-transcriptionally regulate approximately 30% of the human genome [15, 16]. They inhibit the translation, increase cleavage or induce the degradation of target mRNAs depending upon complementary RNA-RNA binding [17]. As miRNAs control a large proportion of the genome, their expression patterns are tissue-specific and dysregulation has been observed in many malignancies [18], suggesting the potential for miRNAs to be biomarkers of cancer diagnosis, prognosis and response to therapies. One gene can be regulated by many miRNAs and likewise one miRNA can regulate several genes, including tumor suppressor genes and oncogenes. This adds an additional layer of functional complexity, as miRNAs can act as both ‘oncomiRs’ to promote tumor development or ‘anti-oncomiRs’ to suppress tumor development, depending upon their tissue expression [19]. Moreover, > 50% miRNA genes are located within fragile sites and genomic regions associated with deletion, translocation and amplification in cancers, further indicating their significance during carcinogenesis [20].

We hypothesised that gastrin may exert some of its pro-tumorigenic effects in the stomach by altering the expression of specific microRNAs, which in turn alter the expression of downstream proteins regulating key cellular processes involved in gastric tumor progression. We have therefore investigated which miRNAs showed altered expression following G17 treatment of a CCK2 receptor expressing gastric epithelial cell line. One of the upregulated miRNAs, miR-222, was further investigated using samples obtained from hypergastrinemic mice and humans and upstream and downstream signalling pathways were defined in AGS_GR_ cells using various inhibitor compounds and siRNA approaches.

## RESULTS

### Gastrin induces miR-222 expression in AGS_GR_ cells

**Figure 1:**
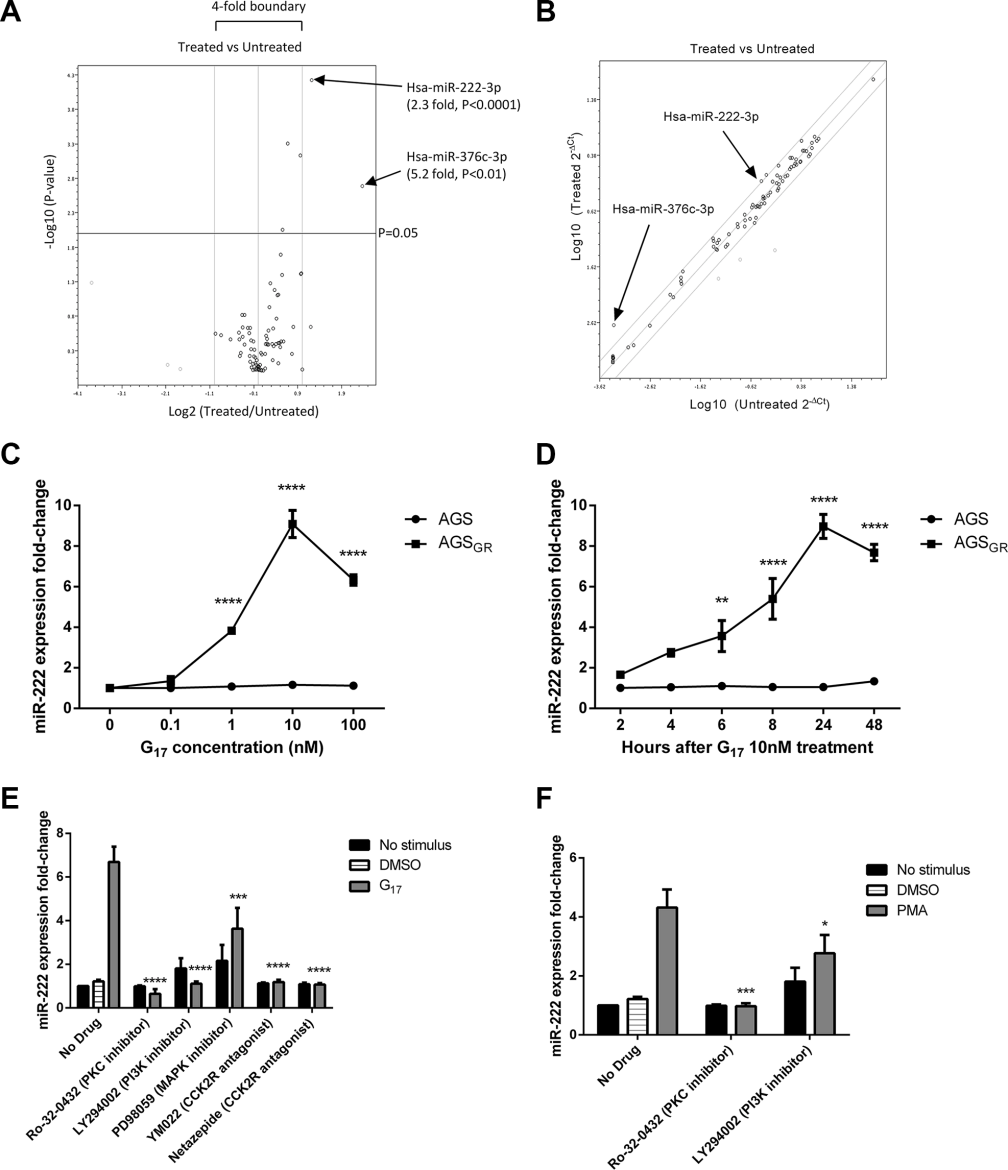
In AGS_GR_ cells treated with 10nM G17 compared with untreated controls, miScript miRNA PCR arrays showed 3 miRNAs that increased and 3 miRNAs that decreased in expression beyond the 2-fold threshold, with only miR-222 and miR-376c proving significant (**A**) As the abundance of miR-376c was low in both the treated and untreated samples, miR-222 was further investigated (**B**). miR-222 expression increased dose and time dependently in AGS_GR_ cells and was maximal following treatment with 10nM G17 for 24h in serum free media. miR-222 expression did not significantly change following G17 treatment of untransfected AGS cells (**C**, **D**). LY294002 (20 μM), YM022 (100 nM), netazepide (100 nM) and Ro-32-0432 (1 μM) all completely reversed while PD98089 (20 μM) partially reversed the miR-222 overexpression caused by 10 nM G17 treatment of AGS_GR_ cells for 24 h (**E**). Ro-32-0432 (1 μM) also completely reversed while LY294002 (20 μM) partially reversed the miR-222 overexpression induced by 100 nM PMA treatment of the same cell line for 24 h (**F**). Statistical significance was determined using two-way ANOVA with Sidak *post-hoc* test and *P* < 0.05 was considered significant. **P < 0.01, ***P < 0.001 and ****P < 0.0001.

miScript miRNA PCR Arrays were used to identify differentially expressed miRNAs between AGS_GR_ cells treated with and without 10 nM G17 for 24 h. Three miRNAs showed increased expression and three miRNAs showed decreased expression beyond the 2-fold threshold (Figure 1A). However, only miR-376c and miR-222 proved significant with fold changes of 5.2 (*P* < 0.01) and 2.3 (*P* < 0.0001) respectively.

Further validation was performed to confirm these differences. As miR-376c was of relatively low abundance in both the control and gastrin-treated cells (Figure 1B) and was also below the threshold for detection by qPCR in this cell line, miR-222 was chosen for further investigation. In parental AGS cells (not stably transfected with the CCK2R), gastrin treatment had no significant effect on miR-222 expression at concentrations of 0–100 nM for 2–48 h. However in AGS_GR_ cells which express the CCK2R, miR-222 expression increased dose and time dependently following gastrin treatment and was maximal after administering 10 nM G17 for 24 h (Figure 1C and 1D).

### Activation of the CCK2 receptor by gastrin leads to increased miR-222 expression via the PKC and PI3K pathways in AGS_GR_ cells

In order to investigate the signalling mechanisms downstream of CCK2R that were responsible for the observed increase in miR-222 expression, we used known inhibitors of these pathways. AGS_GR_ cells were pre-treated with the following signalling pathway inhibitors: Ro-32-0432 (PKC inhibitor), LY294002 (PI3K inhibitor), PD98059 (inhibitor of MAPK activation) and netazepide (YF476) or YM022 (both CCK2R antagonists) prior to treating the cells for 24 h with 10 nM G17. miR-222 expression was evaluated using qPCR primer assays. As miRNAs are regulated by many mRNAs and *vice versa,* miRNAs may be involved in multiple signalling pathways. Small nuclear RNAs, such as the housekeeping gene RNU62 used for normalisation, may therefore also be regulated by the particular pathways investigated. Therefore, an exogenous spike in control (*C.elegans* miR-39) was used for normalisation.

Gastrin-induced miR-222 overexpression was almost fully reversed when AGS_GR_ cells were pre-treated with Ro-32-0432 (1 μM), LY294002 (20 μM), YM022 (100 nM) and netazepide (100 nM) suggesting that miR-222 expression is increased via activation of the CCK2 receptor and subsequent PKC and PI3K pathways. However, there was only a partial reversal caused by the inhibitor of MAPK activation, indicating that this is not the major pathway for miR-222 expression (Figure 1E). Further investigation of downstream signalling was performed by pre-treating AGS_GR_ cells with and without PKC and PI3K inhibitors followed by activation of PKC via PMA 100 nM for 24 h. The activation of PKC stimulated a significant increase in miR-222 expression which was also significantly, but not completely reversed by pre-treatment with the PI3K inhibitor. These data indicate that gastrin-CCK2 receptor activation increases miR-222 expression via both the PKC and PI3K pathways in AGS_GR_ cells (Figure 1F).

### miR-222 expression increases with age in hypergastrinemic INS-GAS mice

**Figure 2:**
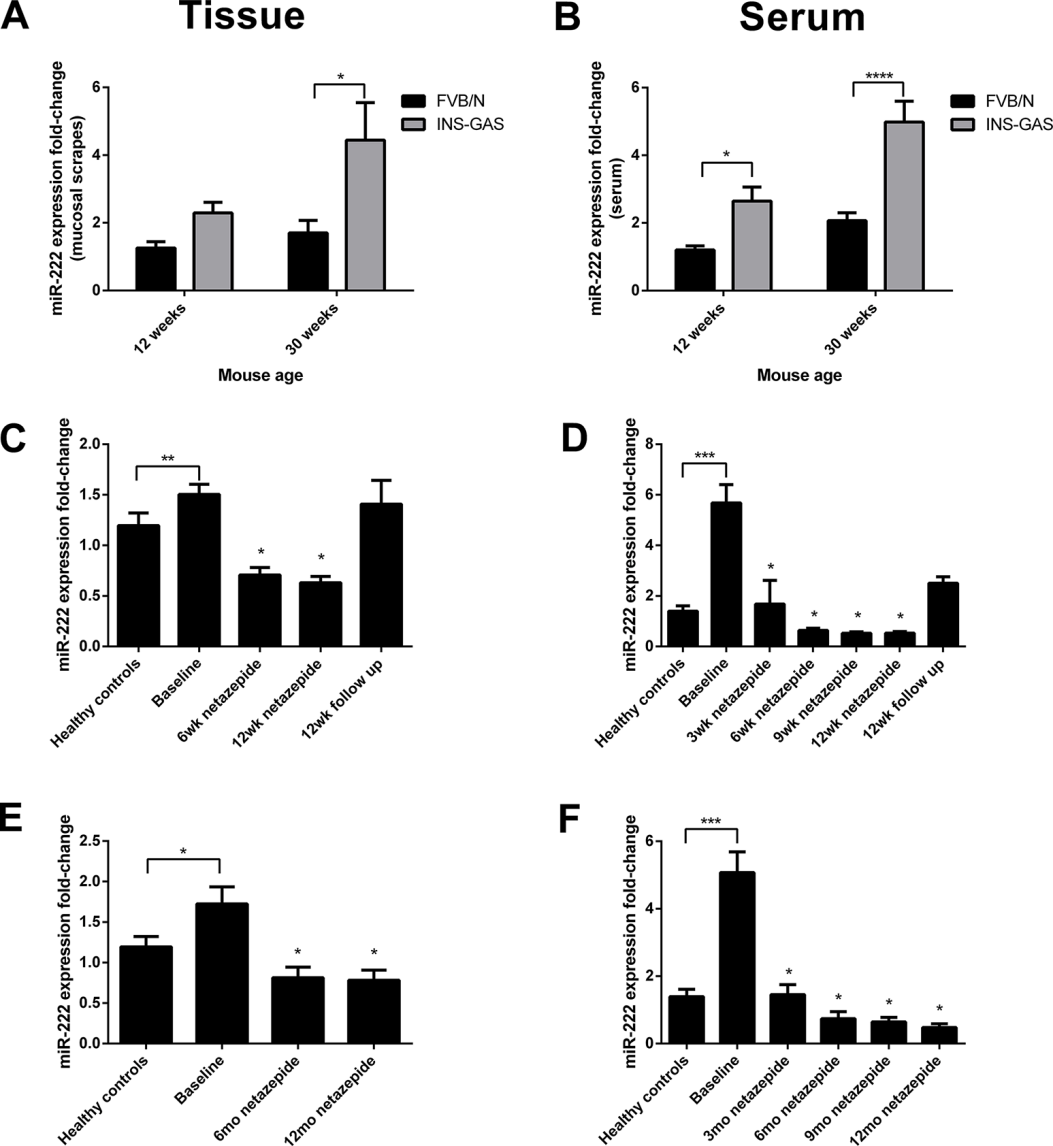
miR-222 expression was significantly increased in mucosal scrapings taken from the gastric corpus (A) and in the serum (B) of 30 week old hypergastrinemic INS-GAS mice relative to 30 week old FVB/N mice, with significant differences also being observed between 12 week and 30 week old INS-GAS mice in the serum (*n* = 10 per group) Statistical significance was determined using two-way ANOVA with Sidak *post-hoc* test and *P* < 0.05 was considered significant *P < 0.05, **P < 0.01 and ****P < 0.0001 between groups. In both gastric corpus biopsies (**C**, **E**) and serum samples (**D**, **F**) from patients with hypergastrinemia and type 1 gastric neuroendocrine tumors (*n* = 8), miR-222 expression was significantly higher at baseline compared with normogastrinemic healthy controls who had a normal stomach at endoscopy (*n* = 10). miR-222 expression decreased whilst patients were taking netazepide and returned to baseline after cessation of treatment, in short (12 weeks, C and D) and long (1 year, E and F) term studies. Statistical significance was determined using a Mann Whitney *U* test between healthy controls and baseline, and by Wilcoxon signed rank test with Bonferroni correction between treatment visits. *P* < 0.0125 was considered significant after correction, *P < 0.0125, **P < 0.001 and ***P < 0.0001.

As previously reported, miRNAs are highly stable in both blood and tissues [24]. miR-222 expression was therefore assessed in FVB/N and transgenic INS-GAS mice on the same genetic background. Mice were culled at 12 and 30 weeks of age (*n* = 10 per group) and gastric corpus mucosal scrapings and serum were used for primer assays. miR-222 expression was significantly increased in both the gastric mucosal scrapings and serum of 30 week old INS-GAS mice compared with age-matched FVB/N wild-type. There was also a statistically significant difference between 12 week and 30 week old INS-GAS mice in the serum (Figure 2A and 2B).

### miR-222 expression is increased in the serum and gastric corpus of patients with hypergastrinemia and type 1 gastric neuroendocrine tumors, and is significantly reduced by netazepide treatment

We also assessed miR-222 expression in both gastric corpus biopsies and serum samples from patients with autoimmune atrophic gastritis, hypergastrinemia and type 1 gastric NETs who had been enrolled on a phase-2 clinical trial to assess the short and long term effects of the CCK2R antagonist netazepide [4]. In addition to the initial 12 week trial, the results of which have been reported, patients were treated for an additional 12 months and showed ongoing histological and biochemical evidence of tumor regression (manuscript in preparation).

There was a significant but small increase in miR-222 expression in the gastric corpus biopsies of hypergastrinemic patients before taking netazepide relative to normogastrinemic controls, which significantly decreased whilst patients were taking 50mg netazepide daily and returned to baseline after cessation of treatment, in both the short-term (12-week treatment with 12-week follow-up) and longer-term (12-month treatment) regimens (Figure 2C and 2E).

miR-222 expression was also significantly increased in the serum of the same hypergastrinemic patients with a 5.7-fold increase in the short-term study and a 5-fold increase in the longer study, when compared to healthy controls. Similarly, whilst patients were taking netazepide, serum miR-222 expression significantly decreased and returned towards baseline after cessation of treatment (Figure 2D and 2F).

### miR-222 overexpression increases migration and the extension of long processes in AGS_GR_ cells

**Figure 3:**
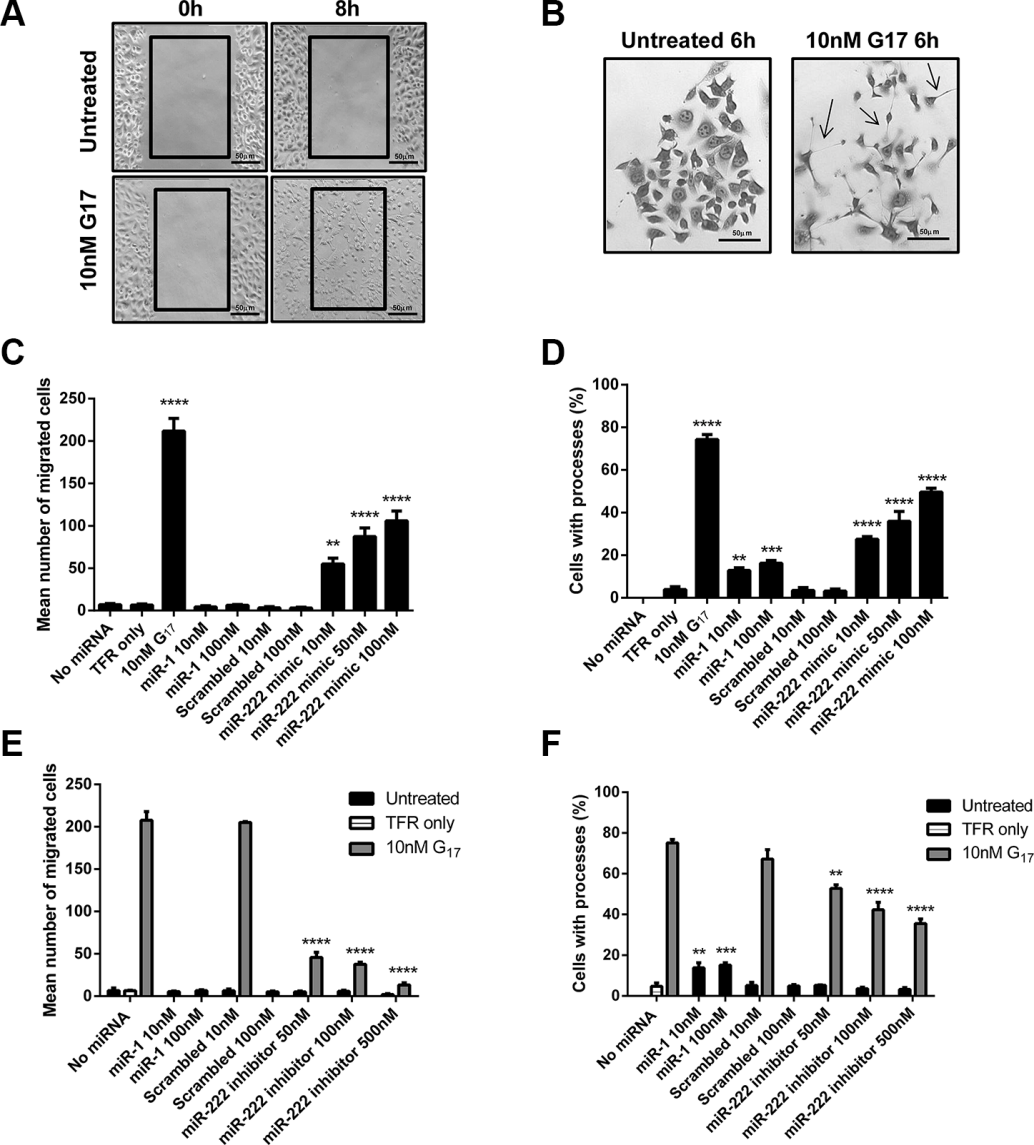
Scratch wound assays were performed to assess cell migration (A) and scattering assays used to assess the expression of long processes (B) following G17 treatment of AGS_GR_ cells Chemically synthesised miR-222 mimics significantly increased migration (**C**) and the extension of long processes (**D**) with statistical significance determined using one way ANOVA with Tukey *post-hoc* test. Whereas miR-222 inhibitors significantly reduced the migration (**E**) and extension of long processes (**F**) that were stimulated by 10 nM G17 treatment of AGS_GR_ cells, in a dose dependent manner and statistical significance was determined using two-way ANOVA with Sidak *post-hoc* test. *P* < 0.05 was considered significant, **P < 0.01, ***P < 0.001 and ****P < 0.0001.

Gastrin has previously been shown to increase the migration of AGS_GR_ cells dose dependently after 8 h treatment with concentrations of 30 pM to 3 nM G17 [22]. Incubation of AGS_GR_ cells with 10 nM G17 for 8 h significantly stimulated cell migration in scratch wound assays (Figure 3A) when compared to untreated AGS_GR_ cells. Chemically synthesised miR-222 mimics at concentrations 10–100 nM also significantly increased AGS_GR_ cell migration dose dependently (Figure 3C). By contrast, chemically-synthesised miR-222 inhibitors significantly reversed 10 nM G17 induced AGS_GR_ cell migration at concentrations 50–100 nM and completely reversed gastrin-stimulated (10 nM) migration at concentration 500 nM (Figure 3E).

Incubation of AGS_GR_ cells with 1nM G17 has also previously been shown to induce cell scattering and the extension of long processes which were maximal after 6 h treatment [23]. Similar responses were observed in AGS_GR_ cells treated with 10nM G17 for 6 h (Figure 3B), with visible morphological changes observed with the extension of long processes (arrows). Chemically synthesised miR-222 mimics dose dependently increased the extension of long processes in AGS_GR_ cells and this was significant at concentrations > 10 nM (Figure 3D). Chemically synthesised miR-222 inhibitors dose dependently reversed the extension of long processes induced by 10 nM G17 in AGS_GR_ cells, which was significant after treatment with concentrations > 50 nM (Figure 3F).

### Gastrin-induced miR-222 overexpression decreases the expression of p27 *in vitro* and *in vivo*, via the PKC and PI3K pathways

**Figure 4:**
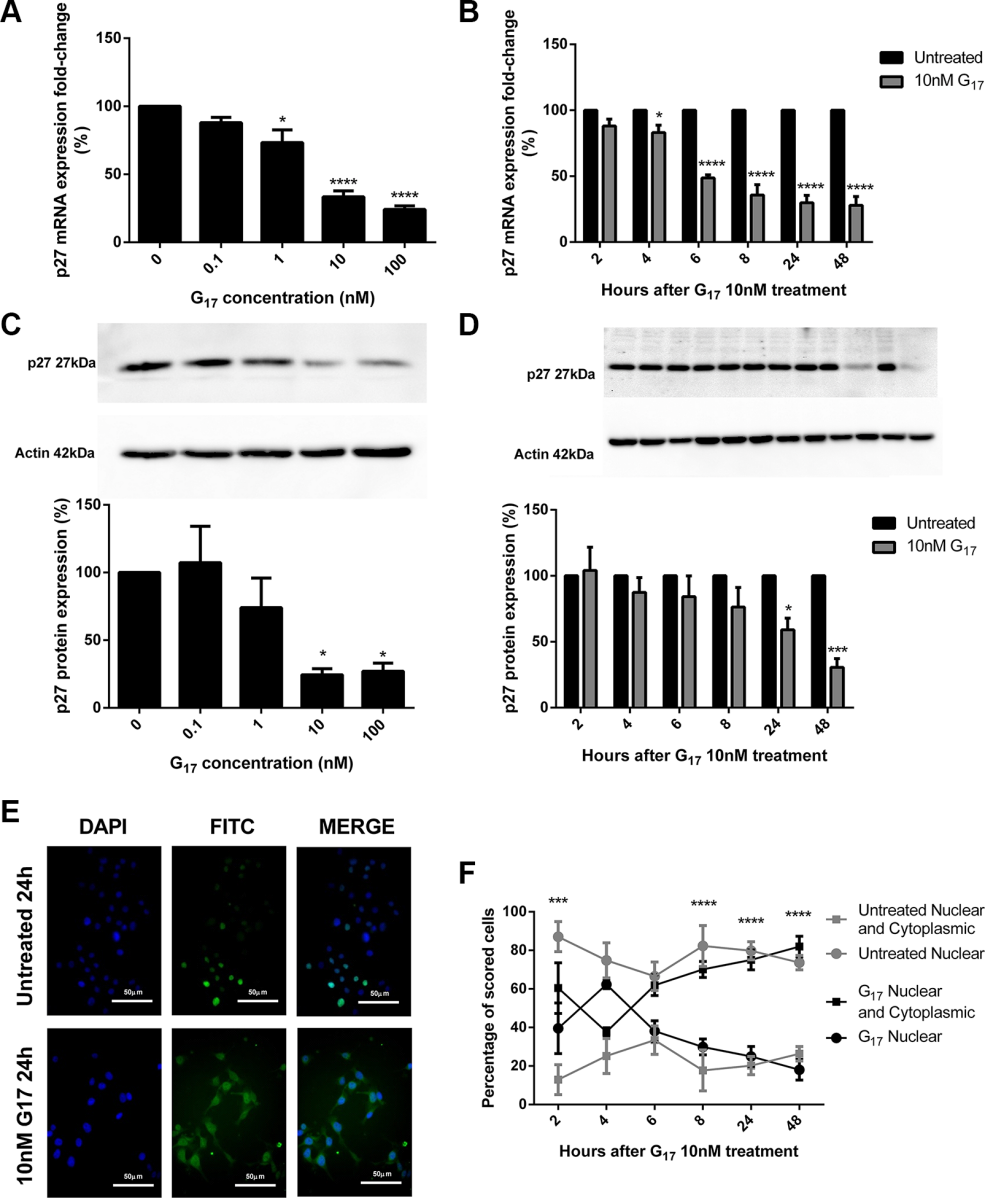
p27 mRNA expression significantly decreased dose (A) and time (B) dependently in AGS_GR_ cells treated with gastrin p27 protein expression dose (**C**) and time (**D**) dependently decreased following gastrin treatment of AGS_GR_ cells. Fold-changes were maximal after 10 nM G17 for 24 h. Representative immunofluorescence images were taken after 24 h treatment with or without 10 nM G17 (×400 magnification) (**E**). 10 nM G17 treatment of AGS_GR_ cells (2–6 hrs) showed the shuttling of p27 with a significant increase in cytoplasmic mislocalisation of p27 after 8 h, in a time-dependent manner (**F**). Statistical significance determined using either one way ANOVA with Tukey *post-hoc* test or two-way ANOVA with Sidak *post-hoc* test and *P* < 0.05 was considered significant.*P < 0.05, ***P < 0.001 and ****P < 0.0001.

In AGS_GR_ cells, qPCR primer assays showed that p27 mRNA expression decreased in dose (Figure 4A) and time (Figure 4B) dependent manners following G17 treatment, which was maximal after 10 nM G17 for 24 h. Western blots indicated that AGS_GR_ cells incubated with G17 also showed dose (Figure 4C) and time (Figure 4D) dependent decreases in p27 protein expression. This was again significant after treating cells with 10 nM G17 for 24 h.

Immunofluorescence visualisation (Figure 4E) showed a dose dependent increase in cytoplasmic abundance of p27 that was significant at a concentration 1 nM and maximal at concentration 10 nM G17 for 24 h. A reciprocal dose dependent decrease in nuclear p27 expression was observed after 0.1 nM G17 for 24 h (data not shown). After 8 h 10 nM G17, there was a significant decrease in the nuclear abundance and increase in the cytoplasmic abundance of p27 in a time-dependent manner (Figure 4F).

**Figure 5:**
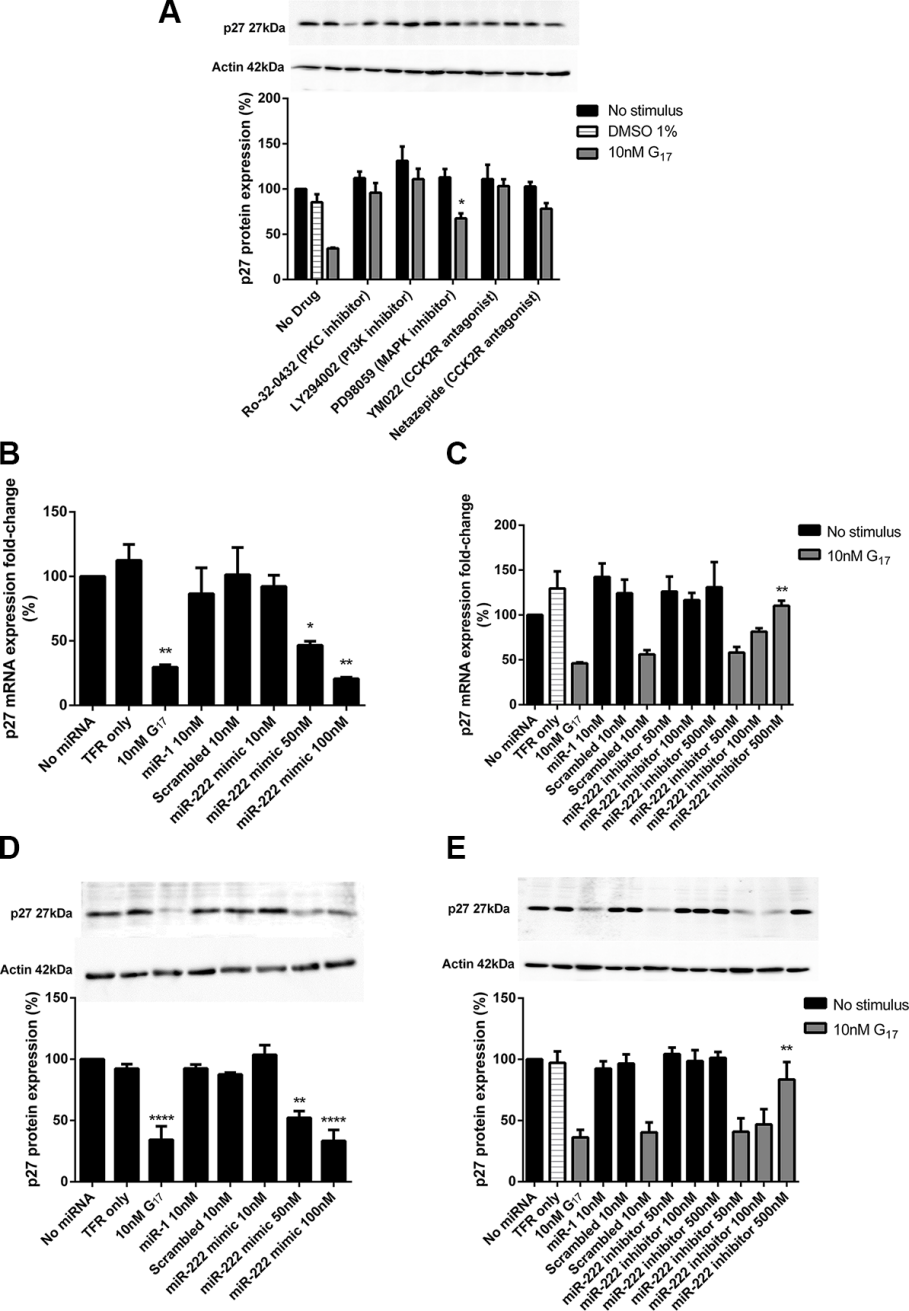
Western blot analysis indicated that LY294002 (20 μM), YM022 (100 nM), netazepide (100 nM) and Ro-32-0432 (1 μM) all completely reversed while PD98089 (20 μM) partially reversed the reduction in p27 expression caused by 10 nM G17 treatment for 24 h (**A**) A chemically synthesised miR-222 mimic significantly reduced p27 mRNA and protein expression (**B**, **D**) whereas a chemically synthesised miR-222 inhibitor partially reversed the reduced p27 mRNA and protein expression that was caused by 10 nM G17 treatment for 24 h (**C**, **E**) in AGS_GR_ cells. Statistical significance was determined using one way ANOVA with Tukey *post-hoc* test or two-way ANOVA with Sidak *post-hoc* test and *P* < 0.05 was considered significant. *P < 0.05, **P < 0.001 and ****P < 0.0001.

The decreased p27 protein expression caused by 10 nM G17 for 24 h was completely reversed by pre-treatment of cells with Ro-32-0432 (1 μM), LY294002 (20 μM), YM022 (100 nM) and netazepide (100 nM) and partially reversed by PD98059 (20 μM) (Figure 5A). AGS_GR_ cells transfected with a chemically synthesised miR-222 mimic showed a dose dependent decrease in p27 mRNA and protein expression (Figure 5B and 5D); this was significant at miR-22 mimic concentrations > 50 nM. The chemically synthesised miR-222 inhibitor at concentrations > 500 nM also partially reversed the decrease in p27 mRNA and protein expression caused by 10 nM G17 (Figure 5C and 5E).

**Figure 6:**
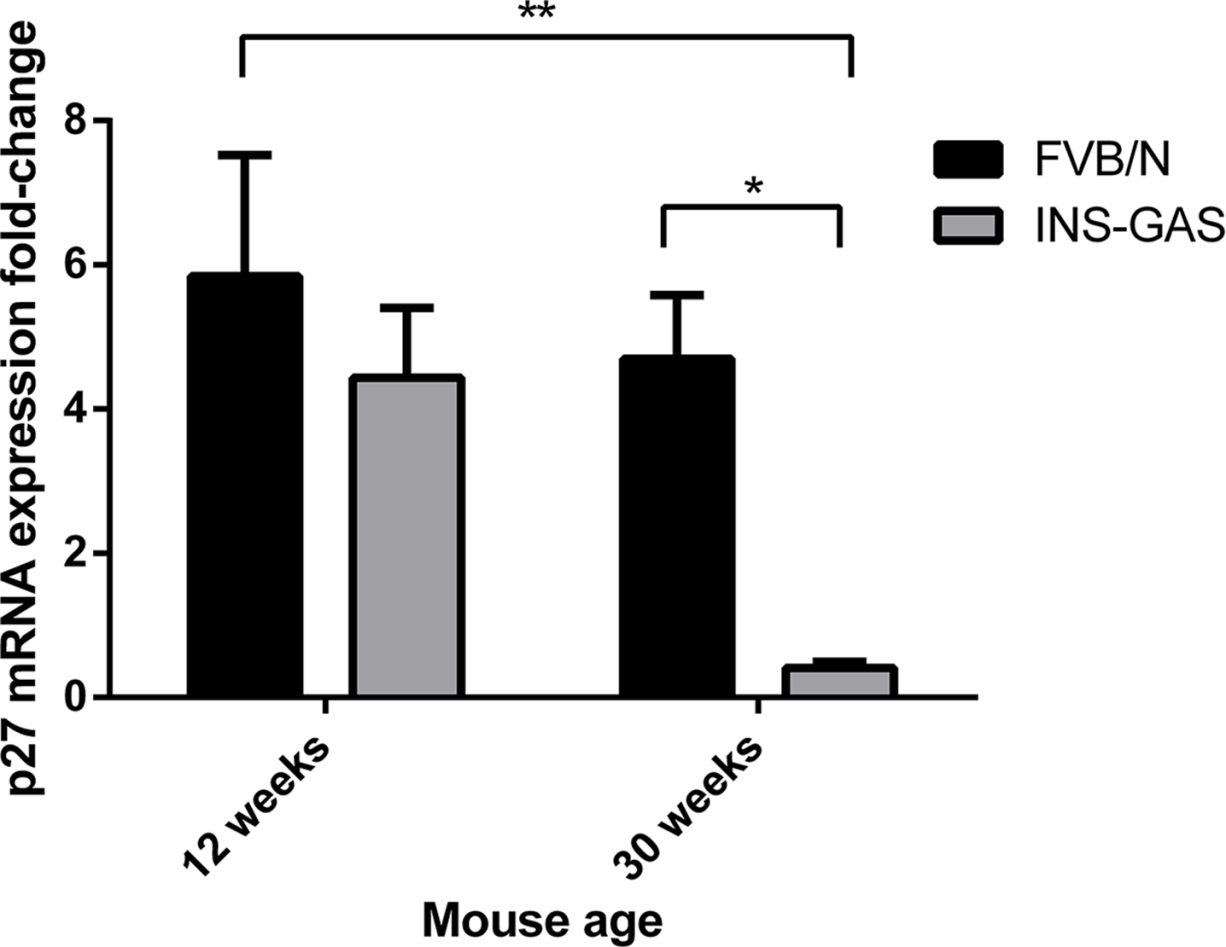
p27 mRNA expression significantly decreased with age in the gastric corpus mucosa of 30 week old hypergastrinemic INS-GAS mice compared with 12 week and 30 week old FVB/N wild-type mice (*n* = 10 per group) (D) Statistical significance was determined using two-way ANOVA with Sidak *post-hoc* test and *P* < 0.05 was considered significant. *P < 0.05 and **P < 0.001.

In 30 week old hypergastrinemic INS-GAS mice, p27 mRNA expression was also significantly decreased relative to age matched FVB/N wild-type mice and 12 week old FVB/N wild-type mice (*n* = 10 per group). There were no significant differences between 12 week old FVB/N wild-type and INS-GAS mice or 12 week and 30 week old INS-GAS mice (Figure 6). These data suggest that gastrin stimulates CCK2R activation leading to increased miR-222 expression which in turn decreases p27 mRNA and protein expression via the PKC and PI3K signalling pathways.

### The reduced p27 expression induced by gastrin increases migration and the extension of long processes in AGS_GR_ cells

**Figure 7:**
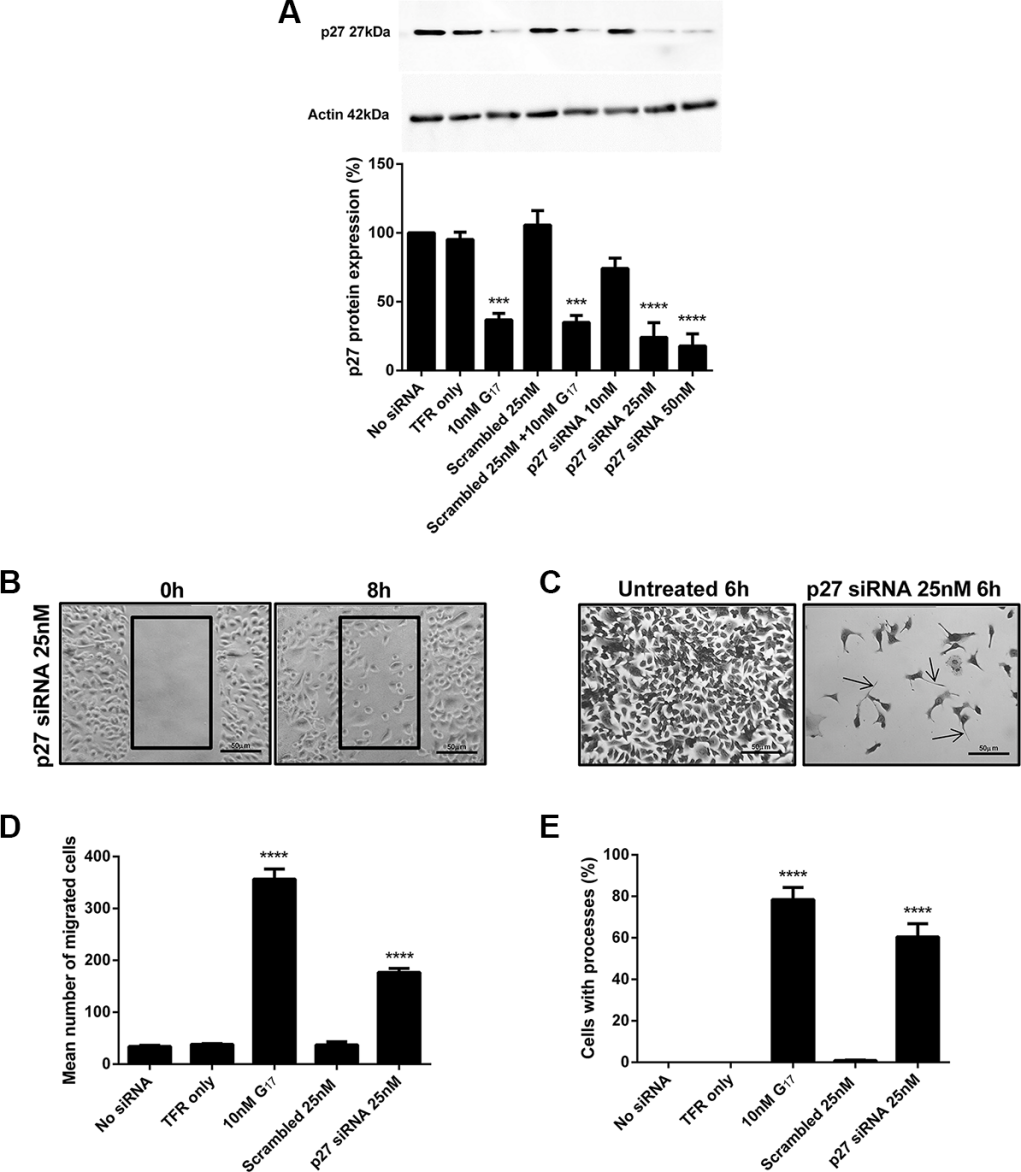
Western blot analysis confirmed that p27 25 nM siRNA knockdown for 48 h resulted in decreased protein expression (A) p27 25 nM siRNA knockdown for 48 h significantly increased cell migration (**B**, **D**) and the extension of long processes (**C**, **E**) in AGS_GR_ cells. Images were taken at×200 magnification. Statistical significance was determined using one way ANOVA with Tukey *post-hoc* test and *P* < 0.05 was considered significant. ***P < 0.001 and ****P < 0.0001.

AGS_GR_ cells were transfected with CDKN1B (p27) siRNA and Western blots confirmed that p27 protein expression was dose dependently decreased after 48 h transfection. The decreased p27 expression was significant following transfection with siRNA concentrations > 25 nM (Figure 7A). The decreased p27 protein expression induced by 25 nM p27 siRNA caused significant increases in cell migration (Figure 7B and 7D) and the extension of long processes in AGS_GR_ cells (Figure 7C and 7E).

## DISCUSSION

**Figure 8:**
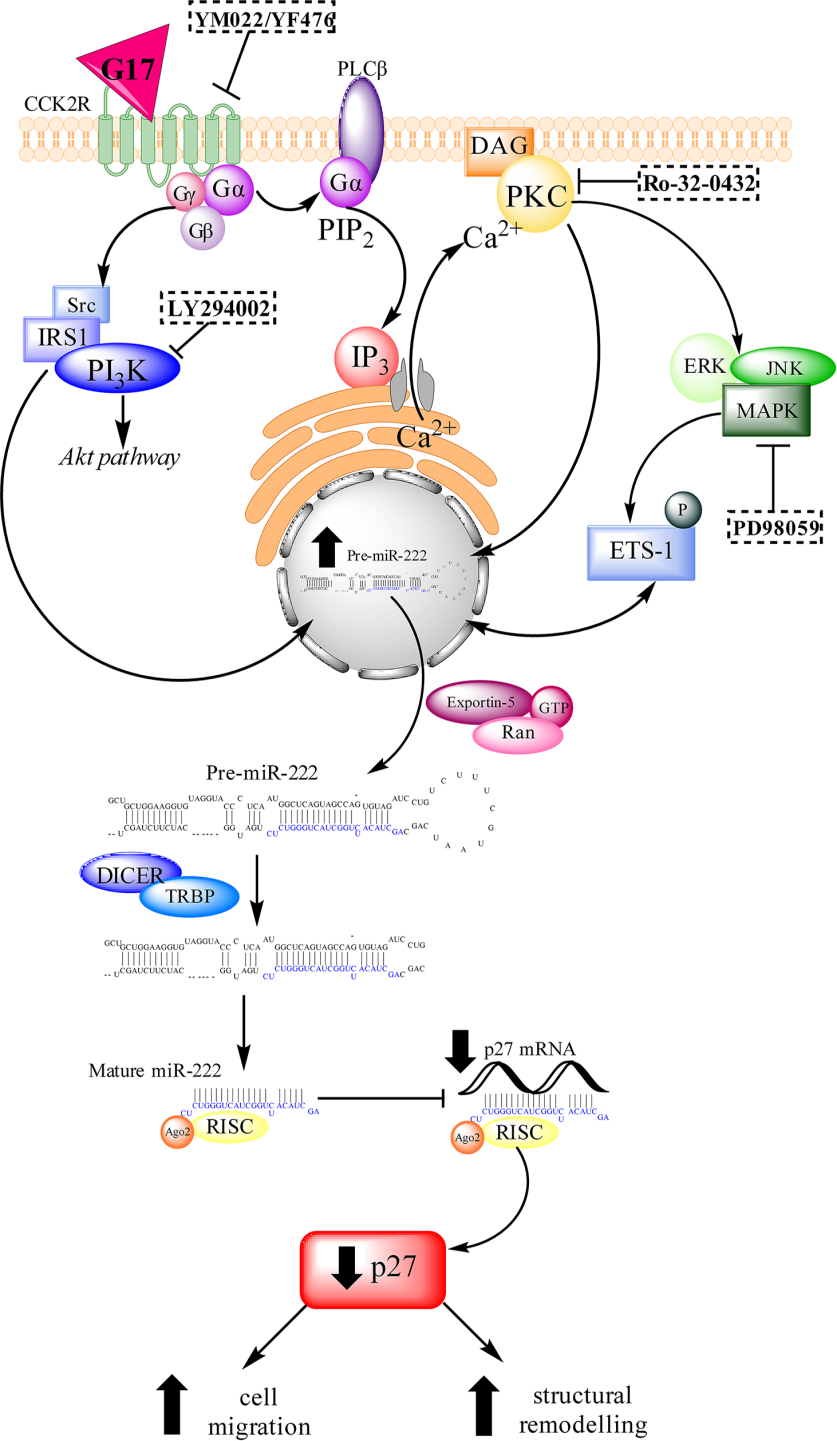
A schematic diagram of the signalling pathways that are activated by gastrin-CCK2R binding miR-222 transcription is increased via the PKC and PI3K pathways and partially via the MAPK pathway. The pri(mary)-miR-222 transcript is cleaved by the RNAse II enzyme Drosha into a hairpin structure (pre-miR-222) which is transported from the nucleus to the cytoplasm via exportin-5. Pre-miR-222 is cleaved by a second RNase II enzyme, Dicer, into mature miR-222 which associates with RISC to target imperfect complementary mRNA sequences. Mature miR-222 inhibits the translation of p27 which increases cell migration and epithelial mesenchymal transition in AGS_GR_ cells.

In summary we have demonstrated that miR-222 expression is increased in AGS_GR_ cells following G17 treatment and that the abundance of this miRNA is also increased in the gastric mucosa and serum of hypergastrinemic mice and humans. Increased gastrin-induced miR-222 expression leads to increased migration and the extension of long processes in AGS_GR_ cells, cellular events which are associated with gastric tumor development. miR-222 exerts these effects at least in part by decreasing p27 expression and causing this protein to be mislocalised in the cytoplasm (Figure 8).

There are few previous reports of the regulation of microRNAs by gastrin. However miR-449 expression was shown to be significantly downregulated in the antrum of gastrin knockout mice relative to wild-type counterparts [25]. The transcription factor E2F1 promotes miR-449 transcription which inhibits the oncogenic genes *CDC25A* and *CDK6*. Reduced expression of *CDC25A* and *CDK6* causes dephosphorylation of the retinoblastoma protein (pRB), which arrests the cell cycle and reduces further E2F1 release [26, 27]. Dysregulation of the miR-449/pRB-E2F1 regulatory loop therefore increases E2F1 activity and promotes cell cycle progression and inhibits apoptosis in gastric cancer. miR-146a expression is also up-regulated in the stomach of gastrin-knockout mice and also in 73% of tested gastric cancers. It targets CARD10 and COPS8, resulting in reduced expression of NF-κB [28].

Several microRNAs have, however, been shown to be dysregulated in gastric adenocarcinoma and in the *H. pylori* infected stomach (reviewed in [29–31]). These miRNAs influence a number of cellular pathways that are involved in gastric carcinogenesis including apoptosis, proliferation and metastasis. Moreover certain miRNAs have also shown promise as biomarkers of both diagnosis and prognosis in gastric cancer [29–31].

Among the miRNAs dysregulated in gastric cancer is miR-222. miR-221 and miR-222 are encoded in tandem from a gene cluster located on chromosome Xp11.3 [32]. Overexpression of miR-222 occurs in several other malignancies including breast, lung, papillary thyroid, prostate and glioblastoma [33–37]. In the GI tract, miR-222 is upregulated in cancers of the esophagus, stomach, colon, liver and pancreas and shows decreased expression in cholangiocarcinoma and gastrointestinal stromal tumors (reviewed in [38]). miR-222 expression is increased in the plasma of patients with gastric cancer relative to patients with chronic active gastritis and healthy controls. Higher levels are associated with more advanced disease [39] and reduced 5 year survival [40]. miR-222 expression is also increased in gastric cancer tissue-derived mesenchymal stem cells [41] and in the stomachs of *H. pylori* infected individuals [42, 43].

miR-222 has several downstream mRNA targets including p27^kip1^, p57, PUMA, PTEN, Bim and MMP1 (reviewed in [44]). Specifically in the stomach, increased miR-222 expression in *H. pylori* infected AGS cells post-transcriptionally regulates RECK and promotes cancer-cell growth and invasion [43, 45]. miR-222 also targets the tumor suppressor PTEN in SGC7901 gastric cancer cells [46]. This negatively regulates the Akt pathway and promotes progression through the cell cycle via the inhibition of p27 and p57 [47]. miR-222 overexpression is also associated with reduced expression of VGLL4 in human gastric cancer cell lines and tissues suggesting that miR-222 inhibits the translation of VGLL4 and promotes YAP-TEAD activation, which is sufficient to increase tumor proliferation, epithelial-mesenchymal transition and invasion [45].

One of the best characterised downstream targets of miR-222 is p27^kip1^ (p27). miR-222 has been shown to bind to the 3′ end of the p27 locus [48]. Several studies have suggested that miR-222-induced inhibition of p27 influences tumor development. There have until very recently been few data linking p27 expression in the stomach specifically to gastrin, but several investigators have previously studied the importance of p27 during *H. pylori* induced gastric carcinogenesis. *H. pylori* infection decreases the expression of the cell cycle inhibitor protein p27 at both transcriptional and post-translational levels. *H. pylori* modulates the G-protein coupled delta opioid receptor (DOR) which regulates the histone acetylation of the p27 gene (CDKN1B) [49]. In addition *cagA+ H. pylori* strains decrease the transcription of p27 through activation of the PI3K/Akt pathway [50]. *H. pylori* infection also promotes the threonine/serine phosphorylation of p27 allowing its accumulation in the cytoplasm [51]. This impairs the ubiquitination of p27 which accelerates its degradation via a proteasome-dependent pathway during cell cycle progression [52]. *p27*-null mice show increased susceptibility to *H. pylori* induced gastric preneoplastic pathology [53]. Patients with *H. pylori-*induced chronic gastritis also show decreased gastric expression of p27, which is reversed following eradication of the bacterium [54, 55]. Long-term exposure to *H. pylori* in animal [56] and cell culture [57] models has also been shown to promote an apoptosis-resistant phenotype associated with decreased p27 expression [58]. A very recent paper has also demonstrated reduced p27 expression in gastric carcinoid tumors arising in transgenic mice that was hypergastrinemic as a result of deletion of Men1 and somatostatin along with treatment with omeprazole [59]. This was associated with alterations in p27 phosphorylation with increases in P-p27^Thr187^, P-p27^Ser10^ and P-p27Kip1^Thr198^ being observed. Moreover this paper also reported loss of p27 expression in human type 1 gastric neuroendocrine tumors associated with atrophic gastritis and showed similar alterations in p27 distribution (by immunocytofluorescence as well as subcellular fractionation) in the CCK2R expressing AGS-E cell line following gastrin treatment [59].

p27 has a number of well described functions that may influence tumor development (reviewed in [60]). When located in the nucleus it is predominantly a cell-cycle inhibitor. The AGS_GR_ cells used in this study show an unusual response to gastrin stimulation with direct inhibition instead of the expected stimulation of proliferation [21]. Therefore we did not consider this cell line to be a good model in which to study the effects of gastrin-induced p27 suppression upon cell-cycle progression. However when p27 islocated in the cytoplasm it also regulates cell migration and invasion in a cell cycle independent manner [60]. Thr^198^ phosphorylation of p27 increases its cytoplasmic localisation, inhibits RhoA (Ras homolog gene family, member A) activation and increases cell motility [61]. Our observation of cytoplasmic mislocalisation of p27 following gastrin treatment is therefore consistent with the increase in migration and change in morphology that was observed in AGS_GR_ cells following treatment with gastrin and a miR-222 mimic.

In conclusion, gastrin induced the expression of miR-222 in CCK2R bearing cells. Increased amounts of miR-222 were also detected in the gastric mucosa and sera of hypergastrinemic patients with type 1 gastric NETs. This miRNA therefore has potential utility as a biomarker of hypergastrinemia and of type 1 gastric NETs. Measurement of serum miR-222 abundance may also be useful for monitoring the response to treatment with CCK2R antagonists such as netazepide. Further work is however needed to investigate whether increased serum miR-222 abundance is specific to patients with type 1 gastric NETs or whether it is also increased in patients who have other causes for hypergastrinemia such as long-term proton pump inhibitor use. Gastrin-induced miR-222 upregulation appears to be functionally important. Increased miR-222 expression results in decreased abundance of p27 mRNA and protein and causes p27 mislocalisation into the cytoplasm. This mechanism appears to contribute to the increased migration and actin remodelling observed in AGS_GR_ cells following G17 treatment (Figure 8). We speculate that similar mechanisms may also be important during the development of gastrin-related gastric tumors *in vivo*.

## MATERIALS AND METHODS

### Reagents

Ro-32-0432, PD-98059, LY-294002 were from Calbiochem (Nottingham, UK), YM022 was from Tocris Bioscience (Bristol, UK), netazepide (YF476) was from Trio Medicines Ltd (London, UK) and amidated G17 was from Bachem (St. Helens, UK). Human miR-222 (MS00007609), miR-376c (MS00004046), RNU62 (MS00033740) and CDKN1B (p27) primers (QT00998445) were from Qiagen (Sussex, UK). Human GAPDH primers were from Eurogentec (Southampton, UK) with the following primer sequences: forward CAGCAAGAGCACAAGAGGAA and reverse GTGGTG GGGACTGAGTGT.

CDKN1B (p27) siRNA and scrambled control siRNA were from GE Dharmacon (Lafayette, USA) as a combination of 4 pooled siRNA sequences (SMARTpool: ON-TARGET*plus*^™^ human CDKN1B (p27) siRNA and SMARTpool: ON-TARGET*plus*™ Non-targeting Pool siRNA) for maximal gene silencing. The CDKN1B (p27) siRNA sequences were as follows: CAAACGUGCGAGUGUCUAA, GCAGCUUGCCCGAGUUCUA, ACGUAAACAG CUCGAAUUA and GCAAUGCGCAGGAAUAAGG. Scrambled control siRNA sequences were as follows: UGGUUUACAUGUCGACUAA, UGGUUUACAU GUUGUGUGA, UGGUUUACAUGUUUUCUGA and UGGUUUACAUGUUUUCCUA. DharmaFECT 1 transfection reagent (GE Dharmacon, Lafayette, USA) was used for siRNA transfection in AGS_GR_ cells. Chemically synthesised miR-222 mimic (MSY0000279), miR-222 inhibitor (MIN0000279), miR-1 mimic positive control (MSY0000416) and miScript negative control (1027271) were all from Qiagen (Sussex, UK). All other routine supplies were from Sigma (Poole, UK) unless otherwise stated.

### Cell lines

The AGS human gastric adenocarcinoma cell line and a transfectant stably expressing the CCK2 receptor (AGS_GR_) were used as previously described [21]. The parental AGS cells were originally obtained from ATCC and both AGS and AGS_GR_ cell lines were confirmed to be mycoplasma-free. Cells were cultured in Ham's F12 medium supplemented with 10% fetal bovine serum (Gibco, Paisley, UK), 2 mM L-Glutamine and 1% combined antibiotics streptomycin and penicillin and maintained in a humidified atmosphere of 5% CO_2_/95% O_2_. All treatments were carried out using serum free media and drug pre-treatments were for 20 mins prior to gastrin treatment.

### Animals

All *in vivo* experiments were carried out on INS-GAS mice on the genetic FVB/N background under UK Home Office project licence numbers 40/3392 and 70/8457, granted after ethical approval. Animals were housed in specific pathogen free facilities with access to food and water *ad libitum* at the University of Liverpool. Gastric corpus mucosal scrapes were obtained after 12 weeks and 30 weeks of age and immediately flash frozen in liquid nitrogen.

### Human samples

Human serum and corpus biopsies were obtained from 8 patients enrolled on a phase-2 clinical trial of the CCK2R antagonist netazepide (YF476) in subjects with autoimmune atrophic gastritis, hypergastrinemia and multiple type 1 gastric neuroendocrine tumors (NETs), as previously described [4]. Control samples were obtained from 10 patients who had a normal upper GI endoscopy, normal gastric antral and corpus biopsy histology, no evidence of *H. pylori* infection by rapid urease test, histology or serology, were not taking proton pump inhibitors and who had fasting serum gastrin concentrations < 40 pM.

### RNA isolation and reverse transcription

Small RNAs were isolated from cells and human biopsies using the miRNeasy Mini Kit and from serum using the miRNeasy Serum/Plasma kit (both from Qiagen, Sussex, UK). Mouse corpus mucosal scrapes were homogenised via bead beating and total RNA was extracted using the High Pure RNA Tissue kit (Roche, Sussex, UK). All extractions were performed according to the manufacturer's supplementary protocols. Eluted RNA was reverse transcribed into cDNA using the miScript RT II Kit (Qiagen, Sussex, UK) according to the manufacturer's procedures handbook and stored as undiluted cDNA at −20°C prior to real-time PCR.

### MicroRNA PCR array

cDNA was prepared using the miScript SYBR Green PCR Kit (Qiagen, Sussex, UK) according to the manufacturer's instructions. The mixture was then loaded into each well of the 96-well miScript miRNA PCR array plate (Qiagen, Sussex, UK) and run in a real-time LightCycler 480 (Roche, Sussex, UK). Data were analysed using the ΔΔC_T_ method of relative quantification and the miScript miRNA PCR array data analysis software (Qiagen, Sussex, UK). Normalisation was against the average threshold cycle of the entire plate minus Ct values > 35 (the maximum threshold value) and 4 control genes (2× miRTC and 2×PPC) that were separately used to assess reverse transcription and PCR performance.

### Primer assays

Mature miRNAs were assessed using primer assays for miR-222 with RNU62 for normalisation according to the miScript miRNA Primer Assay Handbook (Qiagen, Sussex, UK). Messenger RNA was assessed using primer assays for CDKN1B (p27) with GAPDH for normalisation according to the Quantitect Primer Assay Handbook (Qiagen, Sussex, UK) and run in a real-time LightCycler 480 (Roche, Sussex, UK). Each sample was run in quadruplicate, which were analysed by the ΔΔC_T_ method for relative quantification.

### Small interfering RNA (siRNA) transfections

AGS_GR_ cells were transfected with SMARTpool: ON-TARGET*plus*™ human CDKN1B (p27) siRNA (L-003472-00-0005) or SMARTpool: ON-TARGET*plus*™ Non-targeting Pool siRNA (D-001810-10) (both from GE Dharmacon) for 48 h according to the manufacturer's instructions and using DharmaFECT 1 transfection reagent. Cell culture medium was then changed to serum-free medium when 10 nM G17 treatment was applied.

### MicroRNA (miRNA) mimic and inhibitor transfections

AGS_GR_ cells were transfected with either a chemically synthesised miR-222 mimic (MSY0000279) or inhibitor (MIN0000279), a miR-1 positive control (MSY0000416) or miScript negative control (1027271) for 24–72 h according to the manufacturer's instructions and with the use of HiPerfect transfection reagent (301704)(all from Qiagen). Cell culture medium was then changed to serum-free medium when 10 nM G17 treatment was applied.

### Western blot

Protein extracts were prepared and electrophoresed on 12% SDS-polyacrylamide gels, followed by transfer onto nitrocellulose membrane (mdi; membrane technologies INC, Ambala Cantonment, India). Membranes were subsequently blocked in 5% non-fat milk in PBS with 0.1% Tween-20, followed by incubation with the following primary antibodies: mouse monoclonal anti-p27 antibody (BD Biosciences, Oxford, UK) at a dilution of 1:5000 or mouse monoclonal anti-actin antibody (Neomarkers, Freemont, CA) at a dilution of 1:2500. The secondary antibody was horseradish peroxidase (HRP)-conjugated rabbit anti-mouse immunoglobulins (Dako, Cambridge, UK) at a dilution of 1:2000. Membranes were developed using Supersignal (Pierce, Tattenhall, UK) and chemiluminescence was detected using a Bio-Rad ChemiDoc XRS+(Bio-Rad, Hertfordshire, UK). Densitometry was performed using ImageLab software (V 3.0) and results were normalised to actin.

### Immunofluorescence

AGS_GR_ cells were seeded onto 13 mm diameter coverslips (VWR international Ltd, Leicestershire, UK) in 24 well plates (Costar, High Wycombe, UK) at a density of 10^4^/well and left to adhere for 24 h before treatment. After treatment, cells were washed and fixed with 4% paraformaldehyde. Cells were permeabilised with 0.2% PBT (0.03 g BSA, 10 ml PBS and 20 μl Triton-X 100) and blocked in 10% normal horse serum (Vectorlabs, Peterborough, UK) before overnight incubation with mouse monoclonal anti-CDKN1B (p27) primary antibody (diluted 1:500) at 4°C. To detect primary antibodies, the cells were incubated with horse anti-mouse FITC conjugated secondary antibody (Vectorlabs, Peterborough, UK) diluted 1:500 in 1% BSA in PBS for 1h, protected from light. Cells were mounted using Vectashield^®^ media with DAPI (Vectorlabs, Peterborough, UK) onto glass slides for visualisation. Images were captured using an Olympus BX51 fluorescence microscope (Olympus, Sussex, UK) for at least 5 reference fields per treatment at 400× magnification and analysed using ImageJ. Cells were scored as a percentage of total cells that contained nuclear only or nuclear and cytoplasmic p27 staining.

### Cell migration assays

Monolayers of AGS_GR_ cells were grown on 24 well plates in complete media before a cell-free region was created using a 2 μl pipette tip. Cells were washed twice in PBS followed by two washes in serum free media before treatment was applied. Whole cells that had migrated into the denuded region were counted and scratch wound width was measured using a graticule at 0 h and 8 h post treatment [22]. Representative images were taken at these times using a Zeiss Aviovert 25 microscope (Carl Zeiss Microscopy, New York, USA) at x200 magnification.

### Morphological assays

AGS_GR_ cells were seeded in 24 well plates (10^4^/well) in complete media and left to adhere for 24 h before treatment. After treatment, cells were fixed using 3:1 methanol: acetic acid and stained with 0.3% crystal violet. The number of cells that presented long processes were counted as a percentage of total cells in 3 fields per treatment and representative images were taken using the Zeiss Aviovert 25 microscope (Carl Zeiss Microscopy, New York, USA) at×200 magnification [23].

### Statistics

Differences were assessed by either one-way ANOVA with Tukey *post-hoc* test or two-way ANOVA with Sidak *post-hoc* test where appropriate and *P* < 0.05 was considered significant. A Mann Whitney U test was used to assess statistical differences between independent healthy patient samples and baseline samples of patients taking netazepide. A Wilcoxon signed ranked test with Bonferroni correction was subsequently used to determine significant differences between repeated samples from the same patients as not all data were normally distributed and *P* < 0.0125 was considered significant after correction.
